# ThC_2_@C_82_*versus* Th@C_84_: unexpected formation of triangular thorium carbide cluster inside fullerenes†

**DOI:** 10.1039/d2sc04846a

**Published:** 2022-10-20

**Authors:** Yi Shen, Xiaojuan Yu, Qingyu Meng, Yang-Rong Yao, Jochen Autschbach, Ning Chen

**Affiliations:** College of Chemistry, Chemical Engineering and Materials Science, State Key Laboratory of Radiation Medicine and Protection, Soochow University Suzhou Jiangsu 215123 P. R. China chenning@suda.edu.cn; Department of Chemistry, University at Buffalo, State University of New York Buffalo NY 14260-3000 USA

## Abstract

Synthesis of the first thorium-containing clusterfullerenes, ThC_2_@C_*s*_(6)–C_82_ and ThC_2_@C_2_(5)–C_82_, is reported. These two novel actinide fullerene compounds were characterized by mass spectrometry, single-crystal X-ray diffraction crystallography, UV–vis–NIR spectroscopy, and theoretical calculations. Crystallographic studies reveal that the encapsulated ThC_2_ clusters in both C_*s*_(6)–C_82_ and C_2_(5)–C_82_ feature a novel bonding structure with one thorium metal center connected by a C≡C unit, forming an isosceles triangular configuration, which has not been hitherto observed for endohedral fullerenes or for solid phase thorium carbides. Electronic structure calculations assign a formal electronic structure of [Th^4+^(C_2_)^2−^]^2+^@[C_82_]^2−^, with pronounced donation bonding from (C_2_)^2−^ to Th^4+^, secondary backbonding from the fullerene to thorium and Th–C double bond character in both compounds. This work presents a new family of endohedral fullerenes, MC_2_@C_2*n*−2_, being unexpected isomers of MC_2*n*_, and provides broader understanding of thorium bonding.

## Abstract 2

The first thorium-containing cluster fullerenes, ThC_2_@C_*s*_(6)–C_82_ and ThC_2_@C_2_(5)–C_82_, were synthesized and characterized. ThC_2_ clusters in both C_82_ cages feature a novel bonding structure with thorium metal and C≡C forming an isosceles triangular configuration.

## Graphical abstract

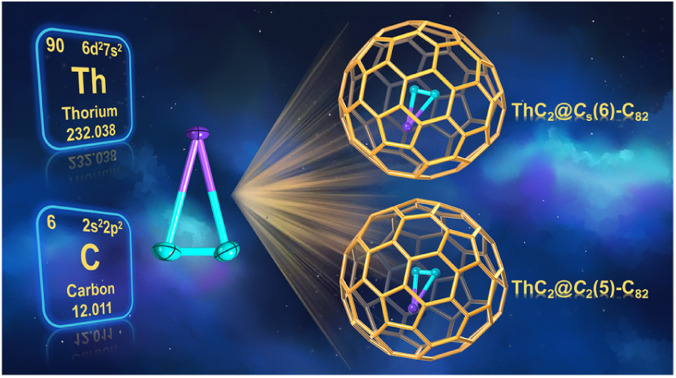

## Introduction

Fullerenes are known for their ability to encapsulate clusters, which results in the formation of unique host–guest molecular compounds—endohedral clusterfullerenes.^1–3^ The unique interaction and mutual stabilization between the metal-containing clusters and fullerenes gave rise to fascinating electronic structures and potential applications of these compounds.^4–7^ To date, most lanthanides have been encapsulated in fullerene cages.^8^ Our recent research showed that novel actinide clusters can also be captured and stabilized by fullerene cages, such as U_2_C@I_*h*_(7)–C_80_, U_2_C_2_@I_*h*_(7)–C_80_, or UCN@C_82_.^9–11^ These systems exhibit substantially different electronic structures compared to known lanthanide-based analogs. In particular, the encapsulated uranium clusters reveal bonding properties that have never been observed in conventional uranium compounds. Thus, the exploration of novel actinide cluster fullerenes will not only expand the scope of endohedral fullerenes, but also have significance regarding the understanding of fundamental actinide chemistry. However, all of the actinide cluster fullerenes discovered thus far were based on uranium; other actinide cluster fullerenes have yet to be explored.^12^

Thorium is arguably the new frontier of nuclear energy.^13^ Attempts have been made to synthesize and characterize thorium compounds for use as potential fuels in advanced reactors. Recently, thorium carbides have attracted increasing attention because these compounds are suitable for high-burnup and high-temperature operations with increased “margin to melting” in the framework of modern nuclear systems.^14^ Many advantages of thorium carbides, such as high melting points, corrosion resistivity, low thermal expansion coefficients and high thermal conductivity, have been reported in recent research.^15,16^ Therefore, understanding the behavior and properties of thorium carbides is essential to explore their potential application as nuclear reactor fuel materials.^17,18^

Thorium carbides (ThC_*n*_, *n* = 1–6) have been detected in vapors above solid carbides or metal alloys in graphite systems, and partial pressures of thorium carbides were measured by mass spectrometry.^19–22^ Thorium dicarbide (ThC_2_), as the main type of stoichiometric thorium carbides, exists in polymorphic modifications at ambient pressure.^16,23–25^ However, the structural and electronic properties of ThC_2_ have only been studied by theoretical calculations.^18,23,26^ Thus far, the molecular structure of ThC_2_ has not been observed in the condensed phase.

On the other hand, carbide cluster fullerenes (CCFs) are an important branch of endohedral cluster fullerenes and have been extensively investigated in the past two decades.^12^ The first reported CCF is Sc_2_C_2_@*D*_2d_(23)–C_84_, initially assigned as a di-metallofullerene (EMF), Sc_2_@C_86_.^27^ This discovery confirmed that the composition M_2_C_2*n*_ could exist as M_2_C_2*n*_ or as M_2_C_2_@C_2*n*–2_. Subsequent studies revealed a large family of CCFs with variable M_2_C_2_ clusters encapsulated inside different fullerene cages, such as Sc_2_C_2_@C_2*n*_,^28–30^ Gd_2_C_2_@C_2*n*_,^31^ Lu_2_C_2_@C_2*n*_^32^*et al.*^2^ In addition, composition Sc_3_C_82_ was also reassigned as Sc_3_C_2_@I_*h*_–C_80_.^33^ Up to now, a large variety of CCFs entrapping multiple (2–4) metal atoms have been reported. However, monometallic carbide cluster fullerenes have not been yet available and whether M@C_2*n*_ can exit as as MC_2*n*_ or as MC_2_@C_2*n*−2_ has yet to be explored.

Herein, we report the first thorium-based cluster fullerenes, namely, ThC_2_@C_*s*_(6)–C_82_ and ThC_2_@C_2_(5)–C_82_. Crystallographic studies reveal that, these two actinide endohedral fullerenes, initially assigned as isomers of Th@C_84_, are in fact thorium-based cluster fullerenes which contains a unique mononuclear thorium carbide cluster. Theoretical analyses confirm that ThC_2_@C_*s*_(6)–C_82_ and ThC_2_@C_2_(5)–C_82_ can be described by a formal two-electron transfer from the ThC_2_ cluster to the C_82_ cage, which results in formal closed-shell electronic structures [Th^4+^(C_2_)^2−^]^2+^@[C_82_]^2−^.

## Results and discussion

### Synthesis and isolation of ThC_2_@C_*s*_(6)–C_82_ and ThC_2_@C_2_(5)–C_82_

**Fig. 1:**
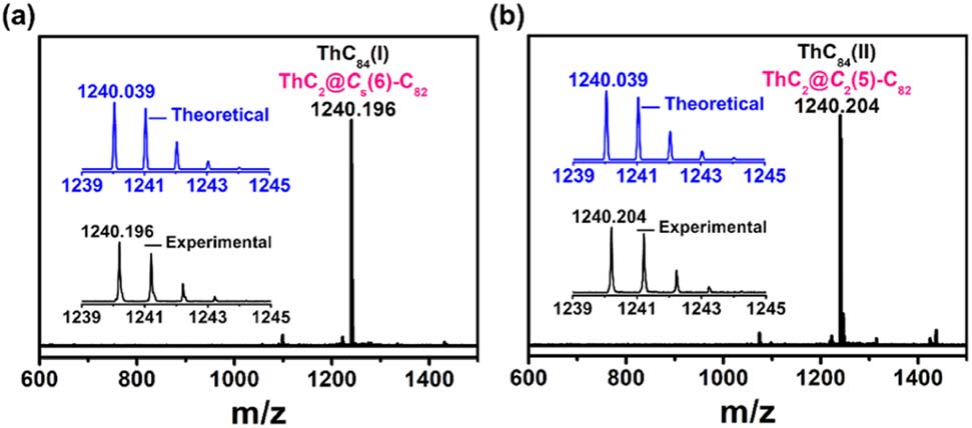
Positive-ion mode MALDI-TOF mass spectra of purified (a) ThC_2_@C_*s*_(6)–C_82_ and (b) ThC_2_@C_2_(5)–C_82_. The insets show the experimental and theoretical isotopic distribution for compounds.

Thorium-based endohedral metallofullerenes were synthesized by a modified Krätschmer–Huffman DC arc discharge method. Graphite rods packed with U_3_O_8_/ThO_2_ and graphite powder (molar ratio of U : Th : C = 1 : 1 : 30) were vaporized in the arcing chamber under a 200 Torr He atmosphere. The resulting soot was then collected and extracted with CS_2_ for 12 h. A series of thorium-containing endohedral metallofullerenes were generated from this process (Fig. S2†) and in this work, two novel isomers(i,ii) of ThC_84_ (later assigned as ThC_2_@C_*s*_(6)–C_82_ and ThC_2_@C_2_(5)–C_82_) were isolated by a multistage high-performance liquid chromatography (HPLC) separation process (Fig. S1†). The purity of the isolated compounds was confirmed by positive-ion-mode matrix-assisted laser/desorption ionization time-of-flight mass spectrometry (MALDI-TOF/MS), as shown in Fig. 1. The mass spectra of ThC_2_@C_*s*_(6)–C_82_ and ThC_2_@C_2_(5)–C_82_ show peaks at *m*/*z* = 1240.196 and 1240.204. In addition, the experimental isotopic distributions of the two samples agree well with theoretical predictions.

### Molecular and electronic structures of ThC_2_@C_*s*_(6)–C_82_·[Ni^II^OEP] and ThC_2_@C_2_(5)–C_82_·[Ni^II^OEP]

Two black block cocrystals were obtained by slow diffusion of Ni^II^(OEP) (OEP = 2,3,7,8,12,13,17,18-octaethylporphin dianion) in benzene into a CS_2_ solution of the corresponding compounds. Ni^II^(OEP) was used to hinder rotation of fullerene molecules in the co-crystal. The molecular structures of ThC_2_@C_*s*_(6)-C_82_·[Ni^II^OEP] and ThC_2_@C_2_(5)-C_82_·[Ni^II^OEP] were determined by single-crystal X-ray diffraction analysis, excluding other molecular structures with the same molecular weight, such as Th-based mono-metallofullerenes, Th@C_84_ isomers. The shortest Ni-cage distance was measured as 2.846(104) and 2.855(115) Å for ThC_2_@C_*s*_(6)–C_82_·[Ni^II^OEP] and ThC_2_@C_2_(5)–C_82_·[Ni^II^OEP], indicating a strong π–π interaction between ThC_2_@C_82_ and Ni^II^(OEP). Both ThC_2_@C_*s*_(6)–C_82_·[Ni^II^OEP] and ThC_2_@C_2_(5)–C_82_·[Ni^II^OEP] were solved in the monoclinic space group C2/*m*.

**Fig. 2:**
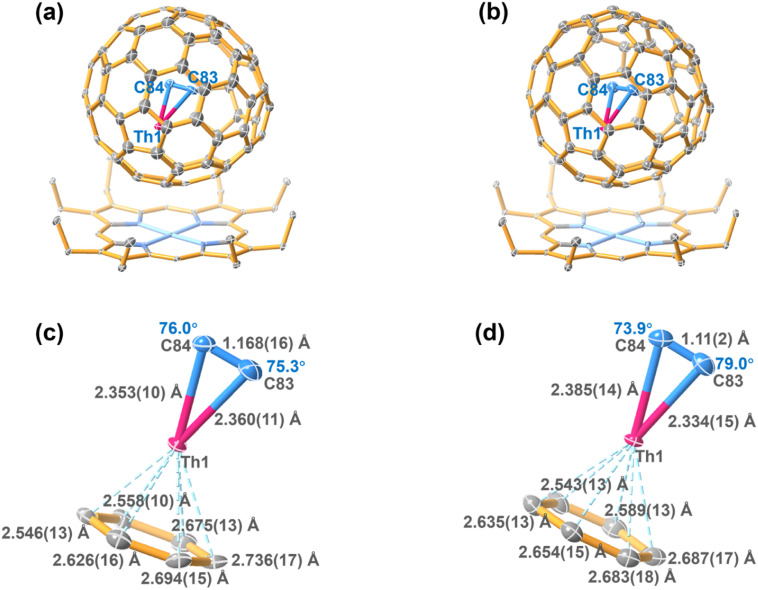
ORTEP drawing of ThC_2_@C_*s*_(6)–C_82_·[Ni^II^OEP] (a) and ThC_2_@C_2_(5)–C_82_·[Ni^II^OEP] (b) with 20% thermal ellipsoids. Only the predominant Th (Th1) sites are shown. For clarity, the solvent molecules and minor metal sites (Fig. S3†) are omitted. Fragment view showing the interaction of the ThC_2_ clusters with the closest aromatic ring fragments of the C_*s*_(6)–C_82_ cage (c) and C_2_(5)–C_82_ cage (d).

The whole molecule of ThC_2_@C_*s*_(6)–C_82_, including the fullerene cage and the encapsulated cluster, shows two equivalent orientations with the same occupancy of 0.5, which is common in many analogous metallofullerene/Ni^II^(OEP) cocrystal systems.^1^ The encapsulated Th ion shows only slight disorder with a total occupancy of 0.5 for the three disordered sites Th1–Th3. Th1 is assigned as the major Th site, as it has a much higher occupancy of 0.418 compared to those of the other two sites (0.0489 and 0.0326 for Th2 and Th3, respectively). Furthermore, Th1A, Th2A and Th3A are also generated *via* their mirror-related counterparts, Th1, Th2 and Th3, due to the same crystallographic mirror plane. Further structural analysis shows that Th1 is situated on the symmetry plane of the C_*s*_(6)–C_82_ cage, while Th1A is located away from the symmetry plane (Fig. S4†). The density functional theory (DFT) calculation results also suggest that the Th1 site is approximately 13 kcal mol^−1^ lower in energy for all functionals than Th1A (Table S2†). In addition, previous studies suggest that the metal ion prefers to remain symmetrically aligned with interacting motifs that share one of the symmetry planes with the fullerene containing mirror planes.^34^ Therefore, we assign Th1 as the optimal position of ThC_2_@C_*s*_(6)–C_82_ (Fig. 2a).

The crystallographic results of ThC_2_@C_2_(5)–C_82_ also show two orientations of the fullerene molecule with equal occupancy of 0.5. These two orientations are related by the molecular crystallographic mirror. The Th1 site is the major Th position for ThC_2_@C_2_(5)–C_82_, with a fractional occupancy of 0.281. Th1A has the same occupancy of 0.281 and is symmetrical with Th1 through a crystallographic mirror. The rest of the minor sites are displayed in Fig. S3(b).† Th1 is located beneath the corresponding hexagon, with the shortest metal-cage distances of 2.542(12) Å (Th1–C3) and 2.589(13) Å (Th1–C2). Its mirror-related counterpart, Th1A, on the other hand, has the closest metal-cage of 2.475(13) (Th1A–C33) and 2.576(14) (Th1A–C55) Å, respectively (Fig. S5†). Thus, in this case, neither Th1 nor Th1A can be assigned as the optimal site only by crystallographic analysis, and their metal-cage distances are very similar. Therefore, theoretical calculations were employed to further determine the optimized position of the encapsulated ThC_2_ cluster relative to the selected cage orientation. The results show that the Th1 site has a lower energy than the Th1A site for ThC_2_@C_2_(5)–C_82_ (Table S2†). Thus, the optimal ThC_2_ cluster orientation can be accurately determined, as shown in Fig. 2b.

As shown in Fig. 2c and d, the Th–C distances are 2.360(11)/2.353(10) Å for ThC_2_@C_*s*_(6)–C_82_ and 2.334(15)/2.385(14) Å for ThC_2_@C_2_(5)–C_82_, which are significantly shorter than the Th–C single bonds in thorium-based organometallic complexes (2.471–2.892 Å),^35,36^ as shown in Table 1. Moreover, for the encapsulated cluster ThC_2_, the C–C distances from the X-ray diffraction are 1.168(16) Å and 1.11(2) Å in the C_*s*_(6)–C_82_ and C_2_(5)–C_82_ cages, respectively, as shown in Table S3.† These C–C bonds can be assigned as triple bonds, but they are approximately 0.1 Å shorter than the optimized distances obtained by theoretical calculations (1.252 and 1.251 Å, respectively) of the isolated cluster fullerenes. The unusual phenomenon of shrinking C–C bonds inside fullerene cages has also been observed for metal carbide cluster fullerenes such as Sc_2_C_2_@*D*_3h_(14 246)–C_74_, Ga_2_C_2_@*D*_3_(85)–C_92_ and U_2_C_2_@*D*_3h_(5)–C_78_, in which the X-ray crystallographically determined C–C bond lengths (1.049(17) Å,^37^ 1.04(2) Å (ref. 38) and 1.127(18) Å (ref. 10) respectively) are also notably shorter than the C–C triple bonds in alkyne compounds (1.21 Å). In addition, as shown in Fig. 2c andd, the metal–C_cage_ distances are 2.546(13)–2.736(17) Å for ThC_2_@C_*s*_(6)–C_82_ and 2.543(13)–2.687(17) Å for ThC_2_@C_2_(5)–C_82_, which agree well with the theoretical calculations (Table S3†) (2.606–2.814 Å for ThC_2_@C_*s*_(6)–C_82_ and 2.635–2.686 Å for ThC_2_@C_2_(5)–C_82_). These distances are similar to the Th–Cp(cent) distances in organometallic compounds; for example, the Th–Cp(cent) distances are 2.532(4)–2.649(8) Å in actinide phosphinidene metallocene (Cp = cyclopentadienyl ring).^39,40^ This result suggests that the coordination interaction between Th and the fullerene cage may be similar to that between Th and the cyclopentadienyl group in organometallic compounds.

**Table 1:** Comparison of Th–C distances in ThC_2_@C_82_ and thorium-based organometallic complexes

system	Th⋯C distance (Å)	Ref.
ThC_2_@C_*s*_(6)–C_82_	2.360(11)/2.353(10)	This work
ThC_2_@C_2_(5)–C_82_	2.334(15)/2.385(14)	This work
[1,3-(SiMe_3_)_2_C_3_H_3_]_4_Th	2.617(5)–2.892(5)	35
[1-(SiMe_3_)C_3_H_4_]_4_Th	2.679(3)–2.806(3)	35
(C_5_Me_5_)_2_ThMe_2_	2.471(8)/2.478(9)	36
(C_5_Me_5_)_2_Th(CH_2_Ph)_2_	2.552(7)/2.551(7)	36

The ThC_2_ cluster in C_*s*_(6)–C_82_ features two almost identical Th–C distances, 2.360(11) and 2.353(10) Å, respectively, leading to an isosceles triangular configuration. ThC_2_ in C_2_(5)–C_82_ has a similar but slightly distorted isosceles triangular configuration, with a Th–C bond length difference of 0.05 Å. Note that the metal–C_cage_ distances in the two ThC_2_@C_82_ isomers, as mentioned already, are also somewhat different [2.546(13)–2.736(17) Å for ThC_2_@C_*s*_(6)–C_82_ and 2.543(13)–2.687(17) Å for ThC_2_@C_2_(5)–C_82_]. This result suggests that the variable isomeric cage structure has a slight impact on the interaction between Th and cage carbon, which likely results in differences in the ThC_2_ cluster configurations inside the two C_82_ cage isomers.

The symmetric isosceles triangular structure configuration of the ThC_2_ cluster encapsulated in either C_2_(5)–C_82_ or C_*s*_(6)–C_82_ is similar to a previously reported theoretically optimized structure of the ThC_2_ molecule:^18^ Kovacs and coworkers predicted that, for neutral ThC_2_, the symmetric triangular arrangement is much more stable than alternate linear or asymmetric triangular conformations.^18,41^ The Th–C distance in the symmetric triangular isolated ThC_2_ molecule obtained in the previous calculations is 2.281 Å,^18,41^ which is shorter than the experimentally obtained Th–C bond length for ThC_2_@C_82_ (2.360(11)/2.353(10) Å for ThC_2_@C_*s*_(6)–C_82_ and 2.334(15)/2.385(14) Å for ThC_2_@C_2_(5)–C_82_). The variability of the Th–C distance may be rationalized by the fact that the coordination bonding between the Th and C_2_ moiety in ThC_2_@C_82_ is weakened by the coordination interaction between Th and the fullerene cage, as discussed later.

**Fig. 3:**
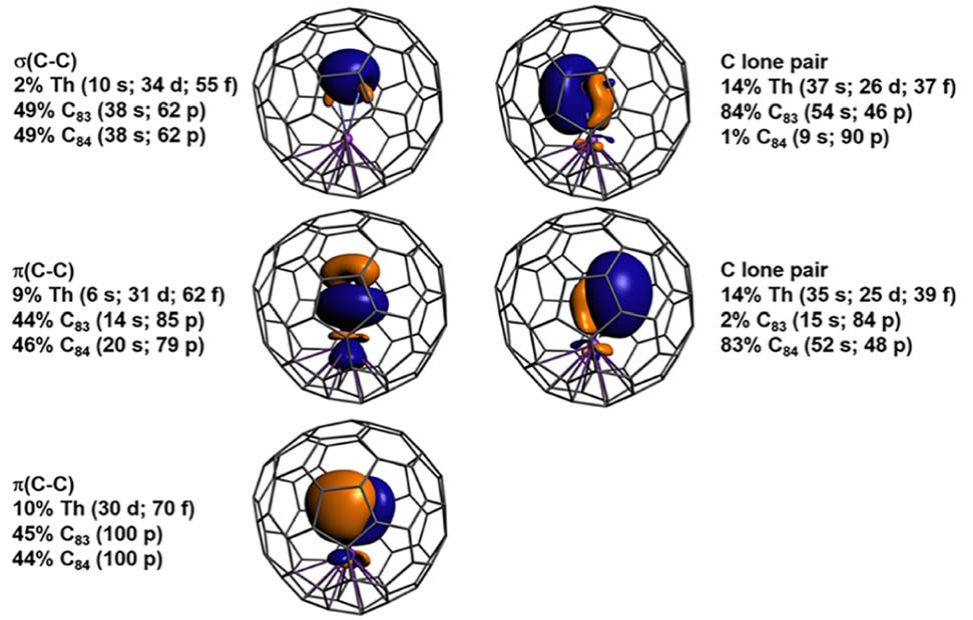
Orbital isosurfaces (±0.03 au) and atomic orbital weight compositions (in %) obtained from NLMO analysis of the singlet ground state of ThC_2_@C_*s*_(6)–C_82_.

**Fig. 4:**
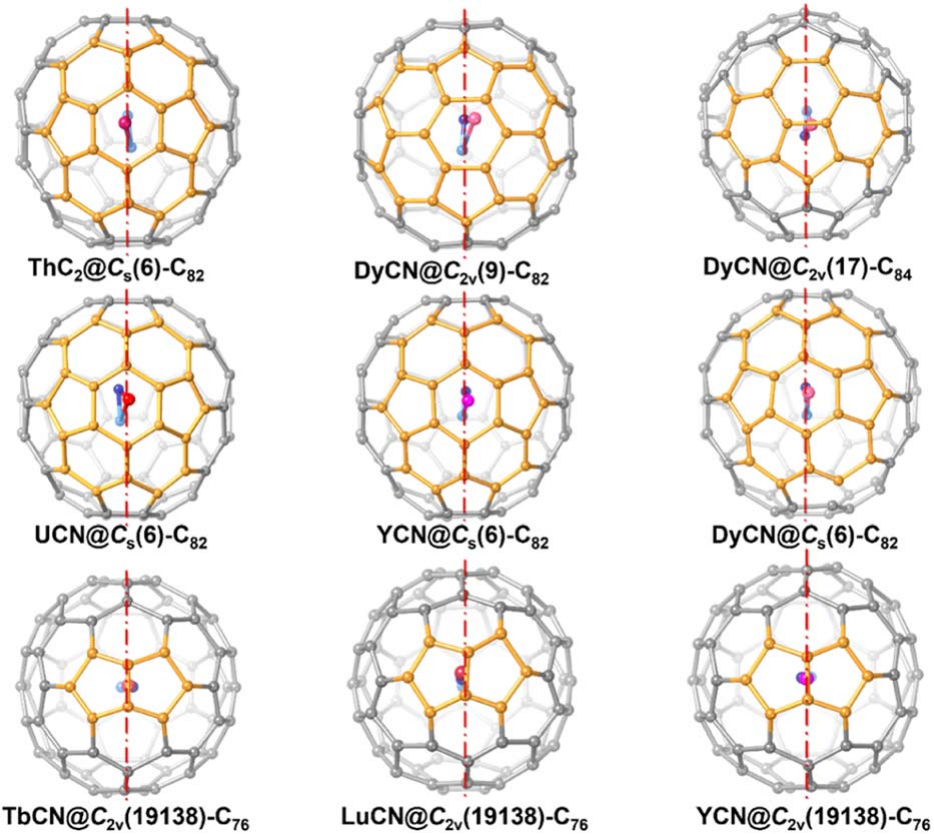
Structures of crystallographically characterized mononuclear cluster fullerenes with pristine cages that contain symmetry planes (highlighted with dotted red lines). The fullerene cage segments closest to the encapsulated metal ions are highlighted in light orange.

A closer look at the symmetric structure of ThC_2_@C_*s*_(6)–C_82_ shows that, although the encapsulated ThC_2_ can have many possible orientations relative to the cage, both the metal atom, Th1, and the cluster, ThC_2_, are located right on the symmetry plane. Further analysis of the crystallographic data of other mononuclear cluster fullerene-containing symmetry planes, such as MCN@C_*s*_(6)–C_82_ (M = U, Y and Dy), MCN@C_2*v*_(19 138)–C_76_ (M = Tb, Lu and Y), DyCN@C_2*v*_(9)–C_82_ and DyCN@C_2*v*_(17)–C_84_, as show in Fig. 4 suggests that the encapsulated mononuclear clusters are all located on the mirror planes of fullerene cages (see Fig. 3).^5,11,42–45^ Previous studies of monometallic fullerenes (only one metal ion inside the cage) have found that in fullerene cages containing symmetry planes, the metal prefers to occupy a symmetric arrangement with respect to the interacting motifs, which share one of their symmetry planes with the fullerene.^34^ This observation further suggests that the endohedral mononuclear cluster also prefers to share a symmetry plane with the fullerene cages. That is, in general, as long as the fullerene encapsulating a mononuclear cluster possesses mirror planes, the entire molecule tends to be symmetric.

The identification of the encapsulated ThC_2_ cluster expands our understanding of endohedral fullerenes. It represents a new type of endohedral cluster MC_2_, in which a single metal ion is coordinated to a C≡C unit. In previous fullerene studies, if a fullerene compound was identified by mass spectrometry as MC_2*n*_, it can be intuitively assigned as a mono-metallofullerene, *i.e.*, M@C_2*n*_, in which only a single metal ion is encapsulated inside the cage. The discovery of MC_2_@C_2*n*_, however, breaks this paradigm and suggests that MC_2*n*_ can also be the isostructural isomer of MC_2_@C_2*n*−2_. Moreover, it provides the first crystallographic observation of a discrete ThC_2_, which may be beneficial for the better understanding of those thorium carbide gas molecules generated in high temperature.

### Theoretical investigation

DFT calculations for ThC_2_@C_*s*_(6)–C_82_ and ThC_2_@C_2_(5)–C_82_ identified spin-singlet ground states. It is known that C_82_ can accept two electrons, for example, in the case of Sm@C_82_ and TbCN@C_82_ [C_*s*_(6) and C_2_(5) isomers].^46,47^ Based on the frontier molecular orbitals in our calculations, we verified that there is a transfer of two electrons from the encapsulated ThC_2_ cluster to the C_82_ cage, *i.e.*, the system adopts a formal closed-shell [Th^4+^(C_2_)^2−^]^2+^@[C_82_]^2−^ electron configuration. Therefore, we interpret that ThC_2_@C_82_ isomers have similar two-electron transfer to those of Sm@C_82_ and TbCN@C_82_. In all three cases, isomeric structures of C_s_(6)–C_82_ and C_2_(5)–C_82_ are stabilized by the metal/cluster-to-cage two electron transfer.^46,47^ For the spin-singlet states, the structural parameters optimized with the B3LYP hybrid functional match the experimental data better than other tested functionals. The following discussion is based on all-electron scalar relativistic B3LYP optimizations and the corresponding electronic structures.

The metal–ligand bonding in ThC_2_@C_*s*_(6)–C_82_ and ThC_2_@C_2_(5)–C_82_, was characterized in terms of natural localized molecular orbitals (NLMOs) and Wiberg Bond Orders (WBOs). In ThC_2_@C_*s*_(6)–C_82_, as shown in Fig. 3, there are three pairs of NLMOs, one σ and two π, describing the formal triple bond of C_2_^2−^. There is pronounced covalency with thorium. The two π NLMOs display three-center characteristics involving Th, with 10% and 9% weights of the orbital density associated with Th 6d–5f hybrids. The carbon lone pairs are even stronger donating, with 13% weight at thorium. The WBO for C_2_^2−^ in the cluster fullerene is 2.51, that is, a triple bond slightly weakened by the donation to the metal. In comparison with other known Th–C bonds, the Th–C interaction in the fullerene shows double bond character. The average WBO of 0.85 is close to the bond order of 0.91 for the formal Th=C double bond in complex [{(NR_2_)_3_}Th(CCCPh_2_)]^−^ (R = SiMe_3_)),^48^ and nearly double the WBOs (0.47 and 0.49) of the single Th–C_*ipso*_ bonds in [Li(DME)_2_(Et_2_O)]_2_[Li(DME)_2_][Th(C_6_Cl_5_)_5_]_3_ and [Li(DME)_2_(Et_2_O)][Li(Et_2_O)_2_][ThCl_3_(C_6_Cl_5_)_3_].^49^ These data help rationalizing the aforementioned short Th–C distances. Some of the NLMOs centered in the fullerene also have density at Th; the corresponding plots are shown in Fig. S9.† Among them, the strongest Th–C(cage) interaction has 6% Th weight. Therefore, the formal transfer of two electrons from ThC_2_ to C_82_ is accompanied by secondary cage-metal backbonding.

The main difference between ThC_2_@C_2_(5)–C_82_ and ThC_2_@C_*s*_(6)–C_82_ (Fig. S8 and S10†) is the backbonding between Th and the fullerene, with only 4% for the largest Th weight in the former, which may rationalize the slightly higher energy of ThC_2_@C_2_(5)–C_82_ by 2 kcal mol^−1^.

### Spectroscopic characterization

**Fig. 5:**
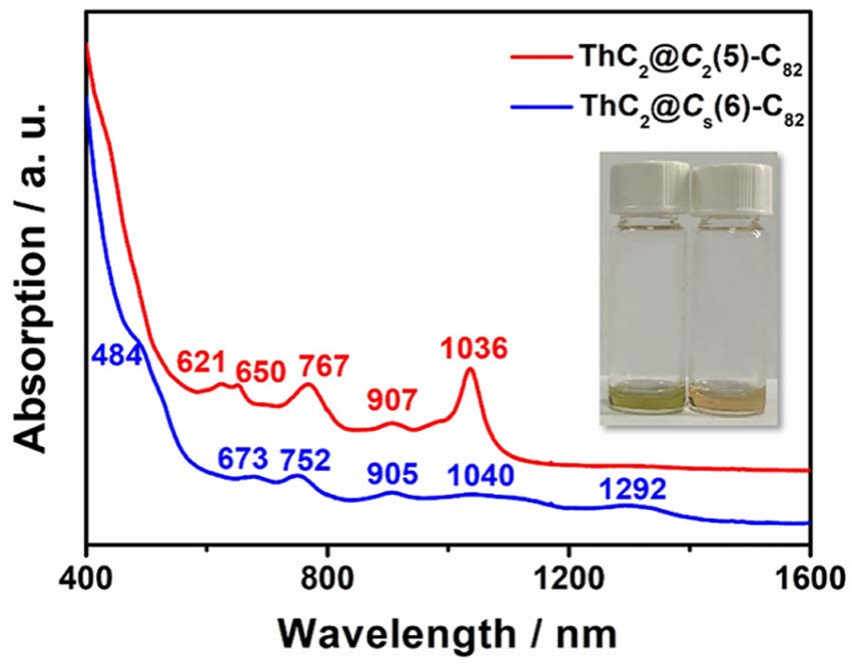
UV–vis–NIR spectra of ThC_2_@C_*s*_(6)–C_82_ and ThC_2_@C_2_(5)–C_82_ dissolved in CS_2_. Insets: Photographs of ThC_2_@C_2_(5)–C_82_ (left) and ThC_2_@C_*s*_(6)–C_82_ (right) dissolved in CS_2_.

The UV–vis–NIR absorption spectra of ThC_2_@C_*s*_(6)–C_82_ and ThC_2_@C_2_(5)–C_82_ dissolved in CS_2_ are shown in Fig. 5. ThC_2_@C_*s*_(6)–C_82_ shows broad peaks at 673, 752, 905, 1040, and 1292 nm and a shoulder peak at 484 nm, similar to those of TbCN@C_*s*_(6)–C_82_.^46^ For ThC_2_@C_2_(5)–C_82_, the characteristic absorption peaks were observed at 621, 650, 767, 907, and 1036 cm^−1^, almost identical to those of TbCN@C_2_(5)–C_82_ with the same fullerene cage.^46^ This indicates similar cage isomer and electronic transfer between ThC_2_@C_82_(C_*s*_(6) and C_2_(5)) and TbCN@C_82_(C_*s*_(6) and C_2_(5)), which is consistent with the results obtained from single-crystal X-ray diffraction and the computational results for [ThC_2_]^2+^@C_82_^2−^.

## Conclusions

For the first time, thorium clusters were encapsulated inside fullerene cages. ThC_2_@C_*s*_(6)–C_82_ and ThC_2_@C_2_(5)–C_82_ were synthesized and characterized by mass spectrometry, single-crystal X-ray diffraction crystallography, UV–vis–NIR spectroscopy and DFT calculations. Crystallographic studies reveal that a mononuclear carbide, which has never been found in endohedral fullerenes, is stabilized inside a C_82_ cage. The two Th–C bond lengths of the ThC_2_ cluster encapsulated in both C_*s*_(6)–C_82_ and C_2_(5)–C_82_ are 2.360(11)/2.353(10) Å and 2.334(15)/2.385(14) Å, presenting isosceles triangular configurations, although the latter shows slight distortion, likely affected by the different cage isomeric structures.

DFT calculations for two isomers of ThC_2_@C_82_ revealed that the electronic structure can be described as a spin singlet ground state, formally [Th^4+^(C_2_)^2−^]^2+^@[C_82_]^2−,^ with pronounced donation bonding from (C_2_)^2−^ to Th^4+^ and secondary backbonding from the fullerene to thorium. The triangular cluster [ThC_2_]^2+^ is more stable in the C_*s*_(6)–C_82_ cage (1a) than in the C_2_(5)–C_82_ cage (2a), which is in part rationalized by a weaker backbonding in the latter. Theoretical analysis also shows a triple bond in the C_2_^2−^ fragment that is somewhat weakened by the donation to the metal. The calculations provide an intuitive description of the bonding of actinide and main group atoms as they are encapsulated in fullerenes.

This work expands the scope of both endohedral fullerenes and actinide compounds. ThC_2_@C_82_ represents a new family of endohedral fullerenes, which reveals for the first time that MC_2*n*_ fullerenes, the most commonly observed endohedral fullerenes, may have two isomeric structures, namely, M@C_2*n*_*versus* MC_2_@C_2*n*−2_. Furthermore, identification of the unique bonding motif of ThC_2_ deepens our understanding of the chemical bonding of thorium.

## Experimental section

### Spectroscopic study

Positive-ion mode matrix-assisted laser desorption ionization time-of-flight (MALDI-TOF) (Bruker, Germany) was employed for mass characterization. The UV–vis–NIR spectra of the purified ThC_2_@C_82_ were measured in CS_2_ solution with a Cary 5000 UV–vis–NIR spectrophotometer (Agilent, USA).

### X-ray crystallographic study

The black block crystals of ThC_2_@C_*s*_(6)–C_82_ and ThC_2_@C_2_(5)–C_82_ were obtained by slow diffusion of the CS_2_ solution of the corresponding metallofullerene compounds into the benzene solution of [Ni^II^(OEP)]. Single-crystal X-ray data of ThC_2_@C_*s*_(6)–C_82_ and ThC_2_@C_2_(5)–C_82_ were collected at 120 K on a diffractometer (Bruker D8 Venture) equipped with a CCD collector. The multiscan method was used for absorption correction. The structures were solved using direct methods^50^ and refined on *F*^2^ using full-matrix least-squares using the SHELXL2015 crystallographic software packages.^51^ Hydrogen atoms were inserted at calculated positions and constrained with isotropic thermal parameters. Crystal data for ThC_2_@C_*s*_(6)–C_82_·[Ni^II^(OEP)]·2C_6_H_6_ and ThC_2_@C_2_(5)–C_82_·[Ni^II^(OEP)]·2C_6_H_6_ are provided in Table S4.†

### Computational details

Kohn–Sham density functional calculations were performed for ThC_2_@C_*s*_(6)–C_82_ and ThC_2_@C_2_(5)–C_82_ structures with the 2017 release of the Amsterdam Density Functional (ADF) suite.^52^ Different functionals, including the Perdew–Burke–Ernzerhof (PBE) and Becke–Perdew (BP86) nonhybrid functional, a global hybrid based on PBE with 25% exact exchange (PBE0), and the popular B3LYP hybrid functionals, were used in conjunction with all-electron Slater-type atomic orbital (STO) basis sets of triple-ζ polarized (TZP) quality for the geometry optimizations and electronic structure analyses.^53–58^ Relativistic effects were considered by means of the scalar-relativistic Zeroth-Order Regular Approximation (ZORA) Hamiltonian.^59^ An atom-pairwise correction for dispersion forces was considered *via* Grimme's D3 model augmented with Becke–Johnson (BJ) damping.^60^ To quantify the compositions of the chemical bonds for selected optimized systems, natural localized molecular orbital (NLMO) analyses were carried out with the NBO program, version 6.0, interfaced with ADF.^61^

## Data availability

CCDC 2183932 and 2183933 contain the supplementary crystallographic data for this paper.† These data can be obtained free of charge *via*https://www.ccdc.cam.ac.uk/data_request/cif. All other data supporting the findings of this study are available from the corresponding authors on request.

## Author contributions

N. C. conceived and designed the experiments. Y. S. and Q. M. synthesized and isolated all the compounds. J. A. and X. Y. performed the computations and theoretical analyses. Y. S. and Q. M. performed the single-crystal measurements. Y. Y. and Y. S. performed the crystallographic analysis. Y. S. performed the spectroscopic measurements. N. C., J. A., Y. S., X. Y., Q. M. and Y. Y. co-wrote the manuscript.

## Conflicts of interest

There are no conflicts to declare.

## Supplementary Material

SC-013-D2SC04846A-s001

SC-013-D2SC04846A-s002
