# Correction: ThC_2_@C_82_*versus* Th@C_84_: unexpected formation of triangular thorium carbide cluster inside fullerenes

**DOI:** 10.1039/d2sc90231a

**Published:** 2023-01-03

**Authors:** Yi Shen, Xiaojuan Yu, Qingyu Meng, Yang-Rong Yao, Jochen Autschbach, Ning Chen

**Affiliations:** a College of Chemistry, Chemical Engineering and Materials Science, State Key Laboratory of Radiation Medicine and Protection, Soochow University Suzhou Jiangsu 215123 P. R. China chenning@suda.edu.cn; b Department of Chemistry, University at Buffalo, State University of New York Buffalo NY 14260-3000 USA

## Abstract

Correction for ‘ThC_2_@C_82_*versus* Th@C_84_: unexpected formation of triangular thorium carbide cluster inside fullerenes’ by Yi Shen *et al.*, *Chem. Sci.*, 2022, https://doi.org/10.1039/d2sc04846a.

The authors regret that incorrect details were given for ref. 53 and 54 in the original article. The correct version of ref. 53 and 54 is given below.

53. J. P. Perdew, K. Burke and M. Ernzerhof, *Phys. Rev. Lett.*, 1996, **77**, 3865–3868.

54. J. P. Perdew, K. Burke and M. Ernzerhof, *Phys. Rev. Lett.*, 1998, **80**, 891.

The authors also regret that there was an error in the sentence in lines 12–13 in the left column on page 5 of the original article. The text originally read, “The carbon lone pairs are even stronger donating, with 13% weight at thorium.” This sentence should read, “The carbon lone pairs are even stronger donating, with 14% weight at thorium.”

The Royal Society of Chemistry apologises for these errors and any consequent inconvenience to authors and readers.

## Supplementary Material

