# Supplementary material for: ThC2@C82versus Th@C84: unexpected formation of triangular thorium carbide cluster inside fullerenes
Source: Chem Sci. 2022 Oct 20;13(44):12980–6. doi: 10.1039/d2sc04846a (PMC9667913; doi:10.1039/d2sc04846a)
Supplement: SC-013-D2SC04846A-s001 [file SC-013-D2SC04846A-s001.pdf]

Electronic Supplementary Material (ESI) for Chemical Science.
This journal is © The Royal Society of Chemistry 2022

Supporting Information

$ThC_2@C_{82}$ versus $Th@C_{84}$: Unexpected Formation of Triangular Thorium Carbide Cluster inside Fullerenes

Yi Shen,[†a] *Xiaojuan Yu,*[†b] *Qingyu Meng,*[a] *Yang-Rong Yao,*[a] *Jochen Autschbach**[b] *and Ning Chen**[a]

[a] College of Chemistry, Chemical Engineering and Materials Science, and State Key Laboratory of Radiation Medicine and Protection, Soochow University, Suzhou, Jiangsu 215123, P. R. China

[b] Department of Chemistry, University at Buffalo, State University of New York, Buffalo, NY 14260-3000, United States

[a] E-mail: chenning@suda.edu.cn

[b] E-mail: jochena@buffalo.edu

Table of Contents

Synthesis and isolation of $ThC_2@C_s(6)-C_{82}$ and $ThC_2@C_2(5)-C_{82}$ S3

Fig. S1. HPLC separation of $ThC_2@C_s(6)-C_{82}$ and $ThC_2@C_2(5)-C_{82}$ S4

Fig. S2. Crystal structures of Th-based mono-metallofullerenes S5

Fig. S3. Disordered thorium and carbon positions in $ThC_2@C_s(6)-C_{82}$ and $ThC_2@C_2(5)-C_{82}$ S5

Fig. S4. The relative position of Th1 and Th1A in $ThC_2@C_s(6)-C_{82}$ S6

Fig. S5. The relative position of Th1 and Th1A in $ThC_2@C_2(5)-C_{82}$ S6

Fig. S6. Structures of crystallographically characterized mononuclear clusterfullerenes containing symmetry planes S7

Fig. S7. DFT optimized structures of $ThC_2@C_{82}$ isomers S8

Fig. S8. NLMO isosurfaces and atomic orbital weight compositions for $ThC_2@C_2(5)-C_{82}$ in the singlet state S9

Fig. S9. NLMO isosurfaces of the carbon cage with Th and atomic orbital weight compositions for $ThC_2@C_s(6)-C_{82}$ in the singlet state S9

Fig. S10. NLMO isosurfaces of the carbon cage with Th and atomic orbital weight compositions for $ThC_2@C_2(5)-C_{82}$ in the singlet state S10

Table S1. Occupancies of disordered thorium sites in $ThC_2@C_s(6)-C_{82}$ and $ThC_2@C_2(5)-C_{82}$ S10

Table S2. Relative energies calculated with different density functionals for $ThC_2@C_s(6)-C_{82}$ and $ThC_2@C_2(5)-C_{82}$. S11

Table S3. Experimental and optimized structural parameters of $ThC_2@C_s(6)-C_{82}$ and $ThC_2@C_2(5)-C_{82}$ S11

Table S4. Crystal structure data of $ThC_2@C_s(6)-C_{82}$ and $ThC_2@C_2(5)-C_{82}$. S12

References S13

Optimized xyz coordinates S13

High-performance liquid chromatography (HPLC) separation process of $ThC_2@C_s(6)$-C_{82} and $ThC_2@C_2(5)$-C_{82}. The first stage was performed on a Buckyprep-M column (25 mm × 250 mm, Cosmosil Nacalai Tesque) with toluene as mobile phase. After that, as shown in Figure S1 (a), fraction from 34 to 36 min (marked in gray) was re-injected into a Buckyprep column (10 mm × 250 mm, Cosmosil Nacalai Tesque) for the second step separation using toluene as the eluent. The fraction marked in orange, which contained two isomers of $ThC_2@C_{82}$ were collected. The third step of separation was conducted on a 5PBB column (10 mm × 250 mm, Cosmosil Nacalai Tesque) using toluene as the eluent. The fraction marked in green, which contained samples were collected and re-injected into the Buckyprep column with a recycle method in the fourth stage. The fraction marked in red contained $ThC_2@C_s(6)$-C_{82} and only a small amount of impurities. Meanwhile, the fraction marked in blue, which contained $ThC_2@C_2(5)$-C_{82} was collected. Figure S1 (b) shows that the MALDI-TOF mass spectrometry of the isolated $ThC_2@C_s(6)$-C_{82} and $ThC_2@C_2(5)$-C_{82}.

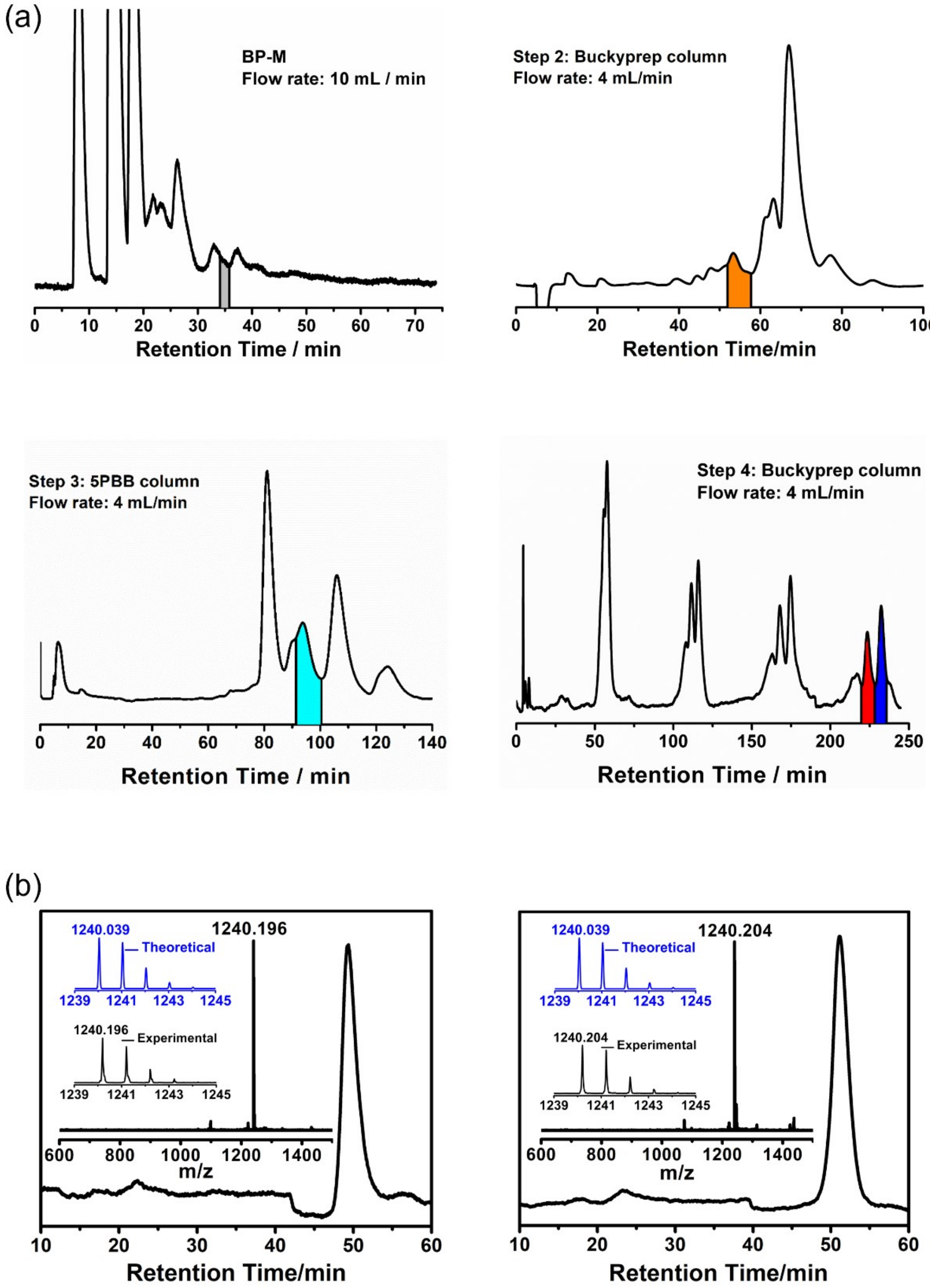

Fig. S1. (a) HPLC profiles showing the separation procedures of $ThC_2@C_s(6)$-C_{82} and $ThC_2@C_2(5)$-C_{82}. (b) HPLC chromatogram of purified $ThC_2@C_s(6)$-C_{82}(left) and $ThC_2@C_2(5)$-C_{82}(right) on a Buckyprep column with toluene as the eluent. (The insets show the positive-ion mode MALDI-TOF mass spectra and expansions of the corresponding experimental isotopic distributions of $ThC_2@C_s(6)$-C_{82} and $ThC_2@C_2(5)$-C_{82} in comparison with the theoretical ones.)

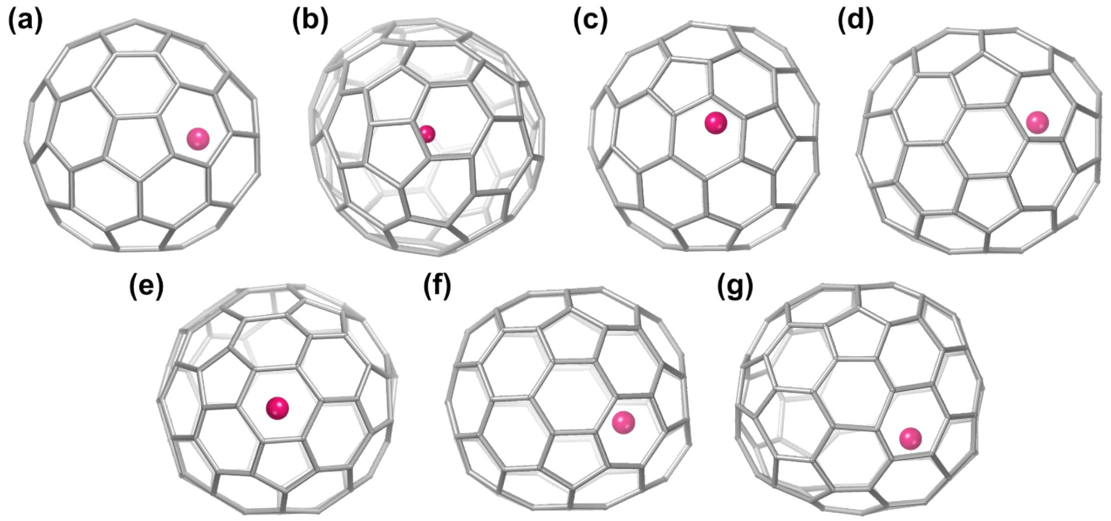

Fig. S2. Thorium-containing endohedral metallofullerenes previously published, e.g. a) Th@T_d(19151)-C_{76},[1] b) Th@C_1(28324)-C_{80},[2] c) Th@D_{5h}(6)-C_{80},[3] d) Th@C_{3v}(8)-C_{82},[4] e) Th@C_2(5)-C_{82},[5] f) Th@C_{2v}(9)-C_{82},[5] g) Th@C_1(11)-C_{86}.[6]

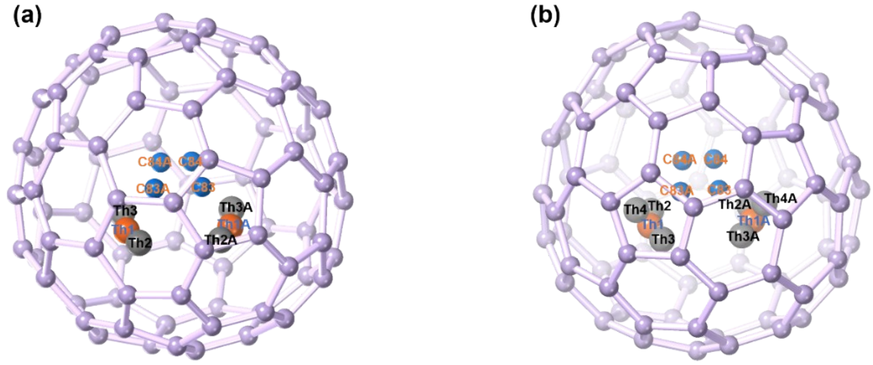

Fig. S3. Drawing of all the Th and C disordered sites. (a) drawing of one orientation of C_s(6)-C_{82} cage together with all the metal disordered sites, six sites for Th are presented, three disordered sites with fractional occupancies of 0.418(2), 0.0489(15), 0.0326(12) for Th1, Th2, Th3, respectively. Another half of the Th disordered sites (Th1A, Th2A and Th3A, respectively) are generated by mirror plane of the crystal. C83A and C84A are generated from C83 and C84 with fractional occupancies of 0.5. (b) drawing of one orientation of C_2(5)-C_{82} cage together with all the Th, C disordered sites, Th1A, Th2A, Th3A and Th4A are generated from Th1, Th2, Th3 and Th4 for which the fractional occupancies are 0.281(3), 0.150(3), 0.0368(17), 0.0327(19), respectively. C83A and C84A are generated from C83 and C84 with fractional occupancies of 0.5.

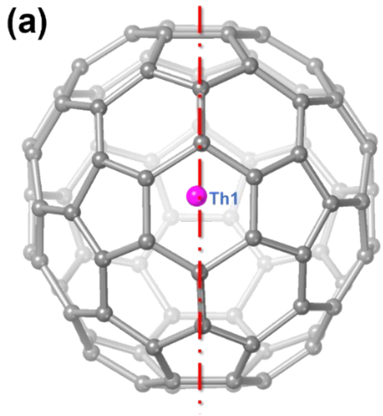

Fig. S4. The relationship of the major Th sites (a) Th1 and (b) Th1A with the Symmetry plane (highlighted with the dotted red line) in $ThC_2@C_s(6)$-C_{82}.

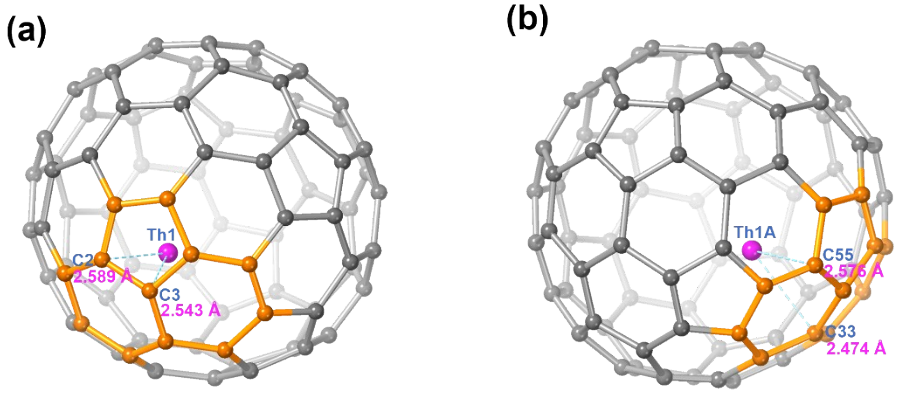

Fig. S5. View showing the interaction of the major Th sites (a) Th1 and (b) Th1A with the closest cage in $ThC_2@C_2(5)$-C_{82}.

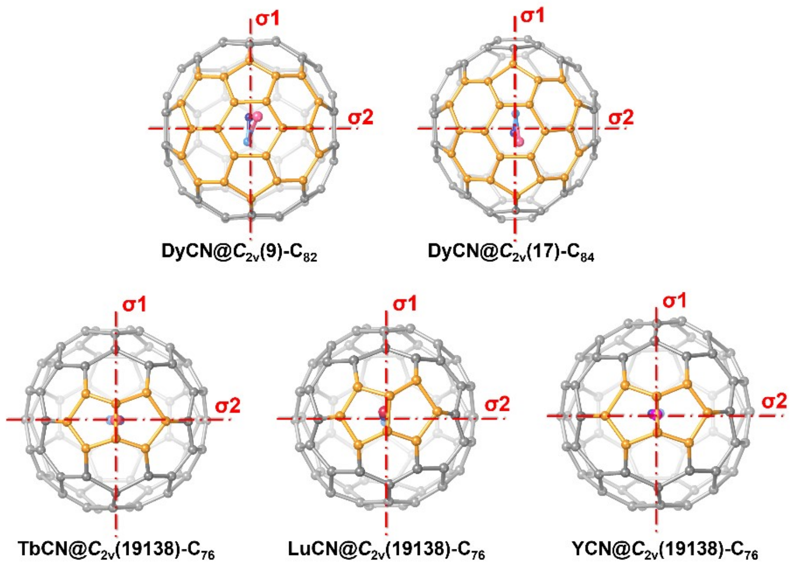

Fig. S6. Structures of crystallographically characterized mononuclear clusterfullerenes with pristine C_{2v} cages that contain two symmetry planes (highlighted with dotted red lines). The fullerene cage segments closest to the encapsulated metal ions are highlighted in light orange.[7-10]

For $ThC_2@C_s(6)$-C_{82} and $ThC_2@C_2(5)$-C_{82}, each of the clusterfullerenes had two conformers depending on the ThC_2 orientation inside the cage, as shown in **Figure S6**, which we labeled **1a**/**1b** for $ThC_2@C_s(6)$-C_{82} and **2a**/**2b** for $ThC_2@C_2(5)$-C_{82}. Because the spin-triplet states are higher in energy by 20.5 and 12.8 kcal/mol than their corresponding spin-singlet states, respectively, the ground spin states for $ThC_2@C_s(6)$-C_{82} and $ThC_2@C_2(5)$-C_{82} are singlets. As seen in Table S2, the global minimum structure is **1a**, which has the lowest energy for all functionals tested. **1b** is 13.5 kcal/mol higher than **1a**; **2a** and **2b** are 2.6 and 10.5 kcal/mol higher in energy than **1a**, respectively. The optimized Th–C distances for **1a** are 2.375 and 2.370 Å, respectively; the two ∠Th–C–C angles are 76.7 and 74.9°, respectively, in good agreement with the experimental data (76.0 and 75.3°). Therefore, we discuss the chemical bonding of conformers **1a** and **2a** (spin-singlet states) as representives in the main text.

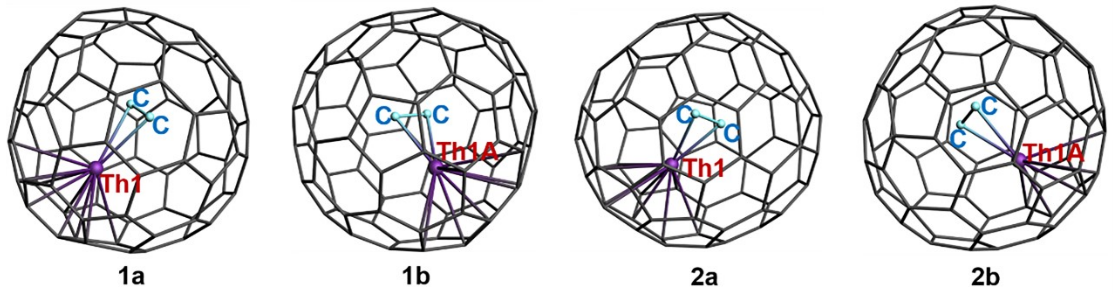

Fig. S7. DFT optimized structures of $ThC_2@C_s(6)$-C_{82} (**1a**/**1b**) and $ThC_2@C_2(5)$-C_{82} (**2a**/**2b**) with different Th sites and two carbon cages in the spin-singlet states.

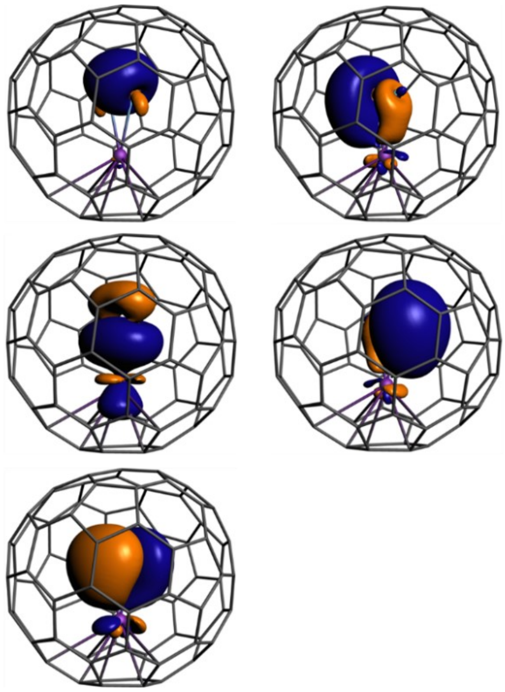

Fig. S8. Orbitals isosurfaces (±0.03 au) and atomic orbital weight compositions (in %) obtained from NLMO analysis of the singlet state of $ThC_2@C_2(5)$-C_{82} (structure **2a**).

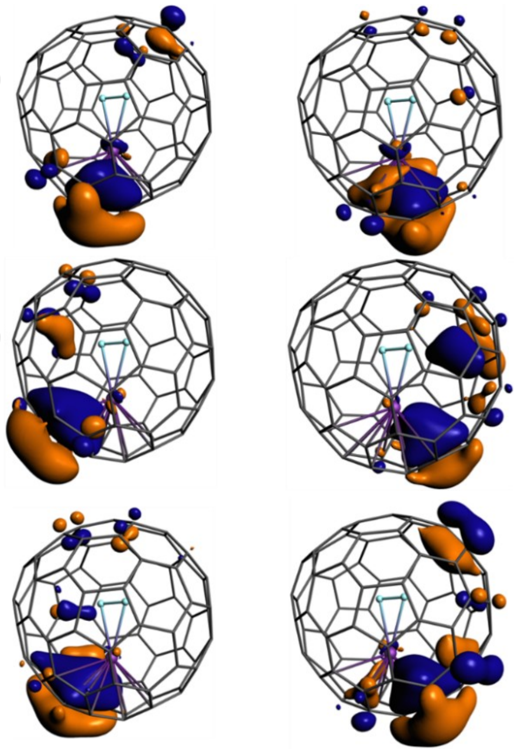

Fig. S9. Orbital isosurfaces (±0.03 au) of the carbon cage with Th and atomic orbital weight compositions (in %) obtained from NLMO analysis of the singlet state of $ThC_2@C_s(6)$-C_{82} (structure **1a**).

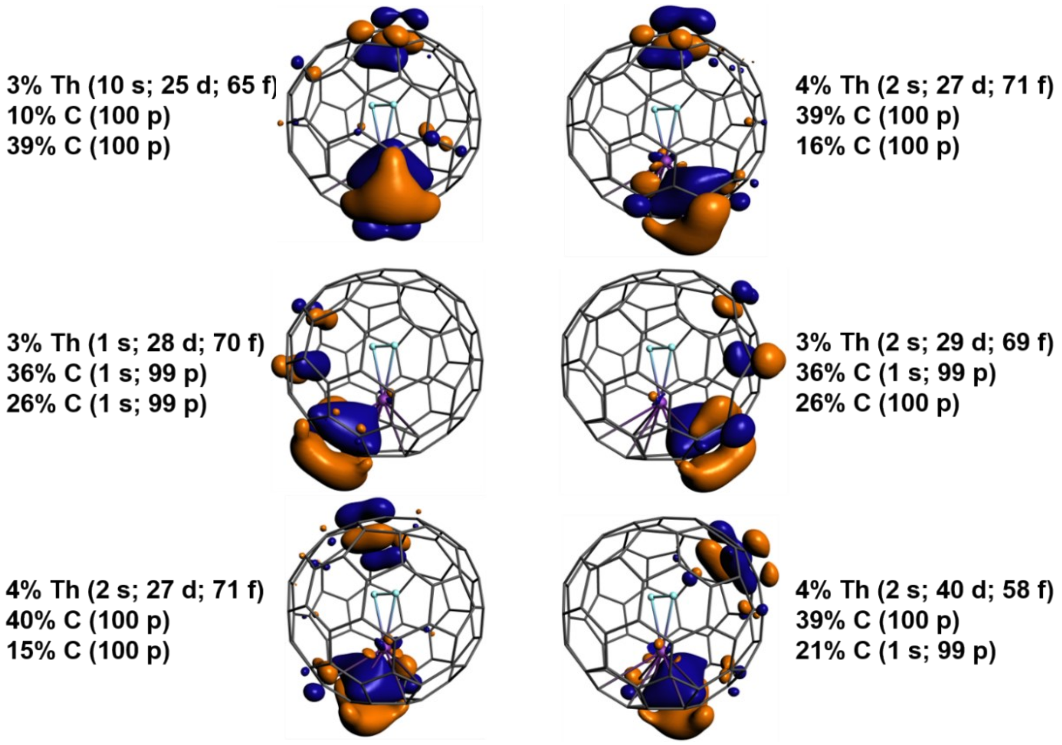

Fig. S10. Orbital isosurfaces (±0.03 au) of the carbon cage with Th and atomic orbital weight compositions (in %) obtained from NLMO analysis of the singlet state of $ThC_2@C_2(5)$-C_{82} (structure **2a**).

Table S1. Occupancies of disordered thorium sites in two isomers of $ThC_2@C_{82}$.

Isomer	Labelling	Occupancy
$C_s(6)$	Th1	0.418(2)
	Th2	0.0489(15)
	Th3	0.0326(12)
$C_2(5)$	Th1	0.281(3)
	Th2	0.150(3)
	Th3	0.0368(17)
	Th4	0.0327(19)

Table S2. Relative energies (kcal/mol) of different isomers of $ThC_2@C_s(6)-C_{82}$ and $ThC_2@C_2(5)-C_{82}$, calculated with different density functionals.

Carbide clusterfullerenes	Sites	PBE	BP86	B3LYP	PBE0
$ThC_2@C_s(6)-C_{82}$	Th1	0.0	0.0	0.0	0.0
	Th1A	14.3	12.2	13.5	15.4
$ThC_2@C_2(5)-C_{82}$	Th1	1.9	1.8	2.6	2.5
	Th1A	8.7	8.5	10.5	11.2

Table S3. Experimental (Expt.) and optimized structural parameters (B3LYP functional) for structures of $ThC_2@C_s(6)-C_{82}$ (**1a**) and $ThC_2@C_2(5)-C_{82}$ (**2a**).

Distance / Å	$ThC_2@C_s(6)-C_{82}$		$ThC_2@C_2(5)-C_{82}$	
	Expt.	Singlet	Expt.	Singlet
Th1-C83	2.360(11)	2.375	2.334(15)	2.367
Th1-C84	2.353(10)	2.370	2.385(14)	2.369
C83-C84	1.168(16)	1.252	1.11(2)	1.251
Th1-C1	2.546(13)	2.617	2.687(17)	2.685
Th1-C2	2.558(10)	2.652	2.589(13)	2.679
Th1-C3	2.675(13)	2.754	2.543(13)	2.660
Th1-C4	2.736(17)	2.857	2.635(13)	2.655
Th1-C5	2.694(15)	2.751	2.654(15)	2.700
Th1-C6	2.626(16)	2.649	2.683(18)	2.702

Table S4. Crystal structure data of $ThC_2@C_s(6)-C_{82}$ and $ThC_2@C_2(5)-C_{82}$.

Crystal	$ThC_2@C_s(6)-C_{82}\cdot[Ni^{II}(OEP)]\cdot 2C_6H_6$	$ThC_2@C_2(5)-C_{82}\cdot[Ni^{II}(OEP)]\cdot 2C_6H_6$
**Formula weight**	1988.55	1988.55
**Crystal system**	monoclinic	monoclinic
**Space group**	*C*2/*m*	*C*2/*m*
***a*, Å**	25.3169(18)	25.261(2)
***b*, Å**	15.0051(11)	14.9722(11)
***c*, Å**	19.9071(15)	19.9879(17)
***α*, deg**	90	90
***β*, deg**	94.325(3)	94.646(4)
***γ*, deg**	90	90
**Volume, $Å^3$**	7540.8(10)	7534.8(10)
Z	4	4
***T*, K**	120(2)	120(2)
**Radiation (*λ*, Å)**	1.34138	1.34138
**Unique data (R_{int})**	7836 (0.0813)	9317 (0.0590)
**Parameters**	1064	1062
**Observed data ($I > 2\sigma(I)$)**	6330	7692
**R_1 (observed data)**	0.0567	0.0773
**wR_2 (all data)**	0.1474	0.2230
**CCDC NO.**	2183932	2183933

REFERENCES

1 M. Jin, J. Zhuang, Y. Wang, W. Yang, X. Liu and N. Chen, *Inorg. Chem.*, 2019, **58**, 16722-16726.

2 W. Cai, L. Abella, J. Zhuang, X. Zhang, L. Feng, Y. Wang, R. Morales-Martinez, R. Esper, M. Boero, A. Metta-Magana, A. Rodriguez-Fortea, J. M. Poblet, L. Echegoyen and N. Chen, *J. Am. Chem. Soc.*, 2018, **140**, 18039-18050.

3 Y. Yan, R. Morales-Martinez, J. Zhuang, Y. R. Yao, X. Li, J. M. Poblet, A. Rodriguez-Fortea and N. Chen, *Chem Commun (Camb)*, 2021, **57**, 6624-6627.

4 Y. Wang, R. Morales-Martinez, X. Zhang, W. Yang, Y. Wang, A. Rodriguez-Fortea, J. M. Poblet, L. Feng, S. Wang and N. Chen, *J. Am. Chem. Soc.*, 2017, **139**, 5110-5116.

5 Q. Meng, R. Morales-Martinez, J. Zhuang, Y. R. Yao, Y. Wang, L. Feng, J. M. Poblet, A. Rodriguez-Fortea and N. Chen, *Inorg. Chem.*, 2021, **60**, 11496-11502.

6 Y. Wang, R. Morales-Martinez, W. Cai, J. Zhuang, W. Yang, L. Echegoyen, J. M. Poblet, A. Rodriguez-Fortea and N. Chen, *Chem Commun (Camb)*, 2019, **55**, 9271-9274.

7 J. Xin, F. Jin, R. Guan, M. Chen, X.-M. Xie, Q. Zhang, S.-Y. Xie and S. Yang, *Inorg. Chem. Front.*, 2021, **8**, 1719-1726.

8 W. Shen, Z. Hu, P. Yu, Z. Wei, P. Jin, Z. Shi and X. Lu, *Inorg. Chem. Front.*, 2020, **7**, 4563-4571.

9 F. Liu, S. Wang, C.-L. Gao, Q. Deng, X. Zhu, A. Kostanyan, R. Westerström, F. Jin, S.-Y. Xie, A. A. Popov, T. Greber and S. Yang, *Angew. Chem. Int. Ed.*, 2017, **56**, 1830-1834.

10 R. Guan, M. Chen, J. Xin, X.-M. Xie, F. Jin, Q. Zhang, S.-Y. Xie and S. Yang, *J. Am. Chem. Soc.*, 2021, **143**, 8078-8085.

Optimized xyz coordinates

Structure **1a** Bond Energy: -19270.15 kcal/mol

C	50.324430	26.510183	55.209046
C	49.272598	27.436266	55.583004
C	48.331631	27.955642	54.600742
C	48.366817	27.554700	53.222295
C	49.412520	26.645386	52.846824
C	50.362777	26.115558	53.814219
C	51.632932	26.017064	53.113284
C	52.901040	26.186726	53.759486
C	52.865283	26.521015	55.148705

C	51.598520	26.729934	55.829153
C	51.837765	27.768223	56.817110
C	50.817758	28.715318	57.141880
C	49.554194	28.539216	56.487954
C	48.800549	29.680239	56.078525
C	48.053576	29.328298	54.915976
C	47.677160	30.304884	53.942341
C	47.490857	29.820235	52.625114
C	47.932177	28.486850	52.257770
C	48.530719	28.558194	50.937259
C	49.711563	27.835662	50.639797
C	50.095454	26.849940	51.600866
C	51.456073	26.456616	51.766924
C	52.510445	27.023789	50.988491
C	53.788772	27.027698	51.598908
C	53.972687	26.657928	52.978568
C	54.976953	27.535014	53.556469
C	54.885209	27.945517	54.910826
C	53.836807	27.388139	55.715143
C	53.201022	28.159970	56.747331
C	53.592604	29.514443	57.009261
C	52.592519	30.429027	57.427290
C	51.197494	30.026398	57.484036
C	50.384343	31.159986	57.125979
C	49.216643	31.021982	56.336023
C	48.859137	32.046137	55.364296
C	48.079565	31.693299	54.168057
C	48.212008	32.509512	53.020157
C	48.059764	31.987558	51.690572
C	47.700889	30.667264	51.493277

C	48.341575	29.889671	50.453307
C	49.312629	30.466780	49.656623
C	50.498411	29.734391	49.308564
C	50.759653	28.440481	49.817459
C	52.161912	28.037281	50.002402
C	53.169454	28.975783	49.679831
C	54.416200	29.030932	50.393266
C	54.727762	28.074916	51.323257
C	55.422240	28.430490	52.542781
C	55.743244	29.780440	52.828169
C	55.769032	30.140029	54.204380
C	55.329169	29.255641	55.217331
C	54.680004	30.043440	56.270941
C	54.724056	31.408207	55.900545
C	53.662800	32.305299	56.205419
C	52.644739	31.801700	57.051954
C	51.278073	32.262074	56.923888
C	50.951616	33.236019	56.017877
C	49.713237	33.170906	55.290977
C	49.950666	33.846149	54.050067
C	49.223458	33.533413	52.943553
C	49.845619	33.499833	51.632481
C	49.064691	32.600047	50.843120
C	49.676627	31.857221	49.849819
C	51.081089	32.000050	49.627019
C	51.551651	30.707336	49.163913
C	52.846060	30.331624	49.349466
C	53.771122	31.222978	50.014257
C	54.768567	30.419603	50.634727
C	55.391928	30.808045	51.842374

C	55.067623	32.112841	52.299491
C	55.081307	32.430596	53.712351
C	55.421003	31.481236	54.631283
C	54.023877	33.379429	53.993829
C	53.310686	33.333789	55.220862
C	51.979337	33.804998	55.162950
C	51.359691	34.150112	53.929168
C	52.017498	33.995488	52.680371
C	51.246272	33.604932	51.491532
C	51.893844	32.818857	50.440433
C	53.304242	32.433392	50.591338
C	54.030503	32.880908	51.712080
C	53.384356	33.665125	52.760998
C	51.390593	30.711215	52.742074
C	52.382790	30.503862	53.476371
Th	50.885050	28.713793	53.914198

Structure **2a** Bond Energy: -19267.56 kcal/mol

C	22.237323	-2.602812	16.146309
C	22.217195	-2.155544	14.771978
C	21.206400	-2.612343	13.828926
C	20.118816	-3.484468	14.241951
C	20.136890	-3.925735	15.617928
C	21.189342	-3.527404	16.539577
C	20.581542	-3.377207	17.837795
C	21.061111	-2.463356	18.831722
C	22.135693	-1.615527	18.466842
C	22.665162	-1.656395	17.110791
C	23.054902	-0.299549	16.766894
C	22.481282	-0.761504	14.474763

C	21.702539	-0.378808	13.349639
C	20.909975	-1.499875	12.959383
C	19.631366	-1.295863	12.343129
C	18.595140	-2.197167	12.691248
C	18.839133	-3.236589	13.682877
C	17.602263	-3.408883	14.426853
C	17.612558	-3.670166	15.818137
C	18.900544	-3.963307	16.375968
C	19.186658	-3.659031	17.733670
C	18.215190	-3.097732	18.624927
C	18.727224	-2.357856	19.712616
C	20.131814	-1.983894	19.778787
C	22.211698	-0.280914	18.954354
C	16.896661	-0.384868	12.606764
C	17.247824	-1.754214	12.804390
C	16.629461	-2.538580	13.843171
C	15.650308	-1.991510	14.646362
C	15.588532	-2.317518	16.039695
C	16.589850	-3.088339	16.679985
C	16.887774	-2.805413	18.093012
C	16.155276	-1.780871	18.738637
C	16.716415	-0.998467	19.800887
C	15.257684	-0.608589	14.460373
C	15.011128	-1.171326	16.687425
C	15.292442	-0.899661	17.989141
C	20.229067	-0.649569	20.291677
C	17.977159	-1.286448	20.286841
C	18.893904	-0.215678	20.620925
C	18.516841	1.095923	20.418263
C	22.867298	0.208312	15.459341

C	21.195533	0.261261	19.796843
C	22.822644	0.514424	17.919488
C	22.464931	1.835257	17.746759
C	22.360638	2.393119	16.431641
C	22.477566	1.600088	15.263797
C	21.698058	1.990502	14.078118
C	21.304597	0.976284	13.104815
C	20.141468	1.148700	12.323676
C	19.272352	0.026034	12.004672
C	17.909385	0.461093	12.090014
C	19.458354	2.042224	19.879338
C	20.797543	1.660818	19.596769
C	21.448881	2.422270	18.597773
C	20.745438	3.385226	17.818023
C	21.373556	3.434430	16.522376
C	20.643938	3.791068	15.432556
C	20.876426	3.139486	14.166073
C	19.664230	3.267289	13.411063
C	19.305891	2.294043	12.498213
C	17.924815	1.872806	12.383710
C	16.970790	2.422182	13.215541
C	15.949147	1.588014	13.792071
C	15.872271	0.204381	13.478662
C	14.902811	-0.091577	15.739718
C	15.501367	0.464806	18.439226
C	16.319448	0.383984	19.603421
C	17.210587	1.403483	19.881116
C	17.345964	2.495825	18.966292
C	18.733634	2.930709	19.025872
C	19.341882	3.570772	17.936453

C	18.535140	3.864634	16.752417
C	19.232024	4.108277	15.550381
C	18.653829	3.853304	14.272799
C	17.343715	3.419584	14.192850
C	16.604280	3.172060	15.392841
C	15.689401	2.075948	15.110220
C	15.224581	1.235632	16.131996
C	15.616777	1.521500	17.511729
C	16.596653	2.579749	17.779898
C	17.160926	3.357351	16.670310
C	18.756122	0.361087	17.017280
C	18.533489	0.642917	15.818476
Th	19.913515	-1.279330	15.760111

1 M. Jin, J. Zhuang, Y. Wang, W. Yang, X. Liu and N. Chen, *Inorg. Chem.*, 2019, **58**, 16722-16726.

2 W. Cai, L. Abella, J. Zhuang, X. Zhang, L. Feng, Y. Wang, R. Morales-Martinez, R. Esper, M. Boero, A. Metta-Magana, A. Rodriguez-Fortea, J. M. Poblet, L. Echegoyen and N. Chen, *J. Am. Chem. Soc.*, 2018, **140**, 18039-18050.

3 Y. Yan, R. Morales-Martinez, J. Zhuang, Y. R. Yao, X. Li, J. M. Poblet, A. Rodriguez-Fortea and N. Chen, *Chem Commun (Camb)*, 2021, **57**, 6624-6627.

4 Y. Wang, R. Morales-Martinez, X. Zhang, W. Yang, Y. Wang, A. Rodriguez-Fortea, J. M. Poblet, L. Feng, S. Wang and N. Chen, *J. Am. Chem. Soc.*, 2017, **139**, 5110-5116.

5 Q. Meng, R. Morales-Martinez, J. Zhuang, Y. R. Yao, Y. Wang, L. Feng, J. M. Poblet, A. Rodriguez-Fortea and N. Chen, *Inorg. Chem.*, 2021, **60**, 11496-11502.

6 Y. Wang, R. Morales-Martinez, W. Cai, J. Zhuang, W. Yang, L. Echegoyen, J. M. Poblet, A. Rodriguez-Fortea and N. Chen, *Chem Commun (Camb)*, 2019, **55**, 9271-9274.

7 J. Xin, F. Jin, R. Guan, M. Chen, X.-M. Xie, Q. Zhang, S.-Y. Xie and S. Yang, *Inorg. Chem. Front.*, 2021, **8**, 1719-1726.

8 W. Shen, Z. Hu, P. Yu, Z. Wei, P. Jin, Z. Shi and X. Lu, *Inorg. Chem. Front.*, 2020, **7**, 4563-4571.

9 F. Liu, S. Wang, C.-L. Gao, Q. Deng, X. Zhu, A. Kostanyan, R. Westerström, F. Jin, S.-Y. Xie, A. A. Popov, T. Greber and S. Yang, *Angew. Chem. Int. Ed.*, 2017, **56**, 1830-1834.

10 R. Guan, M. Chen, J. Xin, X.-M. Xie, F. Jin, Q. Zhang, S.-Y. Xie and S. Yang, *J. Am. Chem. Soc.*, 2021, **143**, 8078-8085.
